# Assessment of Cancer Therapy-Induced Oral Mucositis Using a Patient-Reported Oral Mucositis Experience Questionnaire

**DOI:** 10.1371/journal.pone.0091733

**Published:** 2014-03-10

**Authors:** Anne Margrete Gussgard, Andrew J. Hope, Asbjorn Jokstad, Howard Tenenbaum, Robert Wood

**Affiliations:** 1 Princess Margaret Hospital, Toronto, Ontario, Canada; 2 Faculty of Dentistry, University of Toronto, Toronto, Ontario, Canada; 3 Department of Dentistry, Mount Sinai Hospital, Toronto, Ontario, Canada; 4 Division of Periodontology, Tel Aviv University, Tel Aviv, Israel; 5 Faculty of Health Sciences, UiT The Arctic University of Norway, Tromsø, Norway; Institute of Dentistry, Barts & The London School of Medicine and Dentistry, Queen Mary University of London, United Kingdom

## Abstract

**Objectives:**

Treatment of oral mucositis (OM) is challenging. In order to develop and test useful treatment approaches, the development of reliable, reproducible and simpler methods than are currently available for assessment of OM is important. A Patient-Reported Oral Mucositis Symptom (PROMS) scale was assessed in patients with head and neck cancer to determine if the patient-reported OM experience, as determined by using the PROMS scale, correlate with OM assessed by clinician-based scoring tools.

**Materials and Methods:**

Fifty patients with head and neck cancer and undergoing radiotherapy consented to participate. They were examined before cancer treatment and twice weekly during 6–7 weeks of therapy and once 4–6 weeks after therapy. Signs of OM were evaluated using the 3 clinician-based scoring tools; NCI-CTCAE v.3, the OMAS criteria and the Total VAS-OMAS. The participants' OM experiences were recorded using PROMS-questionnaires consisting of 10 questions on a visual analogue scale. Spearman rank correlation test were applied between the PROMS scale values and the clinician-determined scores. Repeated measures mixed linear models were applied to appraise the strengths of correlation at the different time points throughout the observation period.

**Results:**

Thirty-three participants completed all stages of the study. The participant experience of OM using the PROMS scale demonstrates good correlations (Spearman's Rho 0.65–0.78, p<0.001) with the clinician-determined scores on the group level over all time points and poor to good correlations (Spearman's Rho -0.12–0.70, p<0.001) on the group level at different time points during and after therapy. When mouth opening was problematic, i.e. during the 6^th^ and 7^th^ week after commencing cancer treatment, the Spearman's Rho varied between 0.19 and 0.70 (p<0.001).

**Conclusion:**

Patient experience of OM, as reported by the PROMS scale may be a feasible substitute for clinical assessment in situations where patients cannot endure oral examinations.

## Introduction

**Figure 1:**
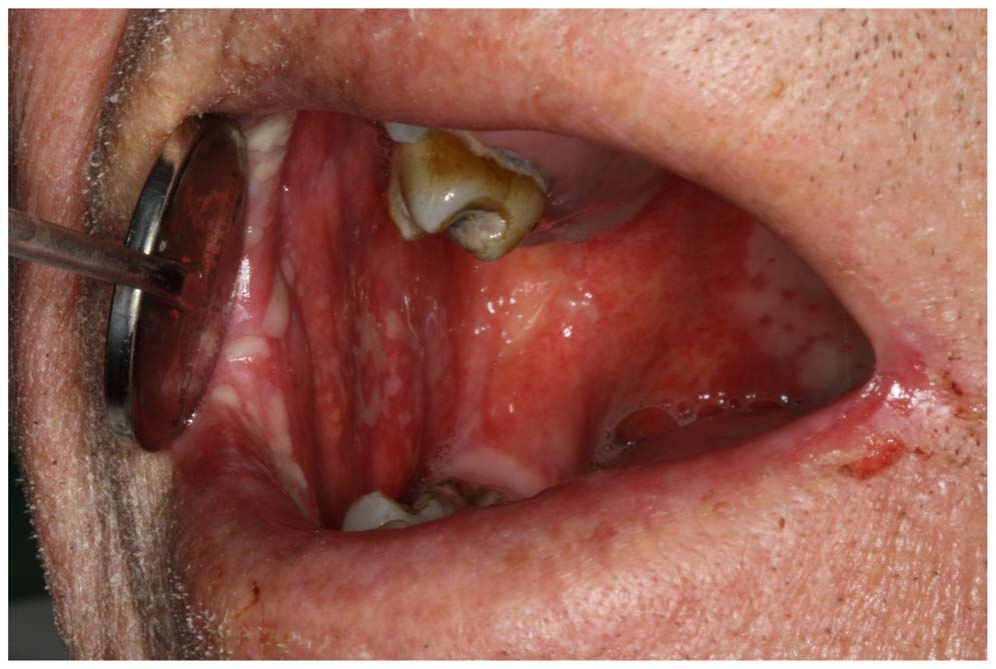
Oral mucositis is a side-effect of radiation treatment that leads to pain and limitations of mouth opening and numerous oral functions.

Numerous clinical studies have focused on mucosal toxicity associated with cancer therapy, which is a common acute toxic effect of radiotherapy in head and neck (H&N) cancer patients [1]–[3]. Oral mucositis occurs in near all patients who receive H&N radiotherapy to the oral cavity or oropharynx and is exacerbated with concurrent chemotherapy [2], [4]. Severe oral mucositis can be very painful leading to decreased intake of food and drink and clinically significant weight loss or dehydration (Fig. 1). Moreover, the psychosocial consequences of debilitating oral mucositis can be considerable since the additional morbidity and pain while undergoing the cancer therapy may cause anxiety and depression [5]–[8]. When severe oral mucositis develops, cancer treatment may be modified or even halted which can limit the efficacy of treatment, and this is estimated to occur in about 10–25% of all patients [9]–[11], although interruption rates as high as 47% have been reported [12]. Severe oral mucositis can lead to increased use of healthcare resources, additional supportive care and even hospitalization. The direct economic consequences of oral mucositis induced by cancer therapies may be significant and require allocation of considerable resources [13]–[15]. Unfortunately, preventing and treating oral mucositis is difficult at best [16], [17]. It is critically important to develop and validate methods that can be used to quantify the oral mucositis experienced by patients in order to develop targeted interventions that efficiently reduce this particular adverse effect of cancer treatment [18].

Extensive resources have been used to find meaningful tools that can be used for accurate assessment of the extent and severity of oral mucositis and/or the burden of oral mucositis for individual patients. Pain associated with oral mucositis is assumed to result from visible ulcerations and from such a perspective it might make sense to use ulceration surface area as a proxy for pain. However, the relationship between size and/or extent of oral lesions and pain is not straightforward and in this regard, other mechanisms of pain experienced by patients with oral mucositis, including neurobiological mechanisms cannot be ruled out [19]. There is a newly emerging body of evidence suggesting that assessments of oral mucositis should include a standardized instrument or a combination of instruments that measure both physical and functional factors, as well as patient-perception [20].

In addition to issues pertaining to assessment of oral mucositis from a clinical perspective (e.g. when and/or if a patient must be provided with less aggressive treatment due to the development of oral mucositis), it has been difficult to assess the efficacy of any particular management protocol for oral mucositis due to the lack of a universally validated and clinically-relevant measurement tool for oral mucositis. Even more importantly, when oral mucositis severity is at its peak, the patient may be unable or unwilling to open his or her mouth to permit a comprehensive clinical assessment of the severity of oral mucositis [21]; a problem that again would interfere with the ability to monitor the condition and also assess the efficacy of various clinical interventions. Hence, in this critical phase of cancer treatment, where a patient may renounce further care, it is critically important to develop other means for assessment of oral mucositis and for confirming the efficaciousness of various treatment interventions for this condition.

Appraising subjective measures that demonstrate a close correlation with intraoral clinical measures may be one strategy. Two promising tools that rely on subjective measurement include the Oral Mucositis Weekly Questionnaire – Head and Neck patients (OMWQ-HN) scale, used in a cohort of head and neck patients [22], and the Patient-Reported Oral Mucositis Symptom (PROMS) scale in a cohort of patients undergoing bone marrow transplantation [23]. The latter measurement tool should be possible for use amongst patients receiving radiotherapy for head and neck malignancy. Hence, a study was designed to evaluate the feasibility of using the PROMS scale to (i) complement common clinician-determined assessments of oral mucositis and (ii) possibly substitute the common clinician-determined assessments of oral mucositis in situations where patients with H&N cancer undergoing treatment have difficulties in opening their mouths for a complete clinical assessment. The hypothesis of this investigation is that the relative magnitude of oral mucositis assessed by clinician-based scoring tools correlates with patient- reported oral mucositis experience as determined by using the PROMS scale.

## Materials and Methods

A prospective single cohort study was designed to appraise the merits of using the PROMS scale to measure how patients with H&N cancer were affected by oral mucositis during their cancer treatment. Approval was obtained from the Research Ethics Boards of the University Health Network (#09-0231-CE) and University of Toronto (# 24171), and written informed consent was obtained from all study participants. The study was conducted at the Princess Margaret Hospital/Ontario Cancer Institute (PMH) in accordance with the ICH Harmonized Tripartite Guidelines for Good Clinical Practice (http://www.pre.ethics.gc.ca/eng/archives/tcps-eptc/Default/).

### Participants

Potentially eligible participants were informed by the dental department staff about the ongoing study. Eligible participants were identified by being 18 years of age or greater and willing and able to provide informed consent. Participation meant a commitment to bi-weekly clinical examination during cancer treatment, and at one postoperative examination. Patients with carcinoma of the oral cavity, nasopharynx, oropharynx, salivary glands or the maxillary sinus scheduled to receive radiotherapy for their cancer with a minimum prescription radiation dose of 54Gy, with or without concurrent chemotherapy were invited to participate in the study. Patients also had a minimum Karnofsky score performance status of 60% and no indications of active significant acute or chronic diseases that might compromise the ability to carry out intraoral assessment of mucositis. Potential participants were advised that at the outset of the study there should be no visible signs of ulcerations. Dental status was appraised as good at the screening visit (no need for dental treatment), fair/poor (dental treatment required before start of cancer treatment) or edentulous. The study recruitment period ended when 50 participants had been enrolled.

### Measures

Participants were scheduled for appointments at baseline, twice weekly over the course of their 6 to 7-week cancer treatment and once more 4 to 6 weeks after completion of treatment. At each appointment participants had an oral examination by a previously-calibrated investigator with the help of mouth mirrors and the use of a high-power head-lamp as a light source. Participants reported how oral mucositis which developed during the radiotherapy period impacted on selected oral functions using the PROMS-questionnaire. Analgesic use, need for hospital admission, or the addition of nutritional support since the previous examination was recorded based on self-reports provided by the participants.

### Clinical oral examination

Clinical signs of oral mucositis were recorded using three different clinician-based scoring tools, two of which are probably the most common tools used by clinicians worldwide, i.e., the clinical component of the National Cancer Institute Common Terminology Criteria for Adverse Events version 3 (NCI-CTCAE v. 3) [24], and the clinical component of the Oral Mucositis Assessment Scale (OMAS) [21]. The third tool has been developed locally and is termed “TOTAL-VAS-OMAS” [23]. In the NCI-CTCAE v. 3 the occurrence and severity of oral mucositis is graded using an ordinal score ranging between 0 (none) and 4 (most) as observed at any site within the oral cavity. The OMAS tool was used as described previously whereby a score of 0 (none) and 3 (ulceration) or 2 (erythema) is assessed in nine specific intra-oral locations. The ulceration and erythema scores were not aggregated as in the original publication, but kept separate to better elucidate possible correlations with the other clinician-based scoring tools and the PROMS experience. Hence, the maximum sum score of ulceration was 27 (9 sites x3) and of erythema 18 (9×2). The “TOTAL-VAS-OMAS” tool consists of two visual analogue scales ranging between 0 to 100 mm for full mouth assessments of erythema and ulceration respectively. The first author (A.M.G.) undertook training and calibration in oral mucositis assessment prior to initiation of the study until Kappa  = 1.0, by the use of a photographic set developed for such purposes for the OMAS tool, kindly provided by Dr. Monique Stokman at the University Medical Center Groningen, The Netherlands. Additionally, a laminated booklet containing these images was used during the study to maintain reliability. Most participants made strong efforts to allow complete assessment of their oral conditions, despite the presence, for example, of severe oral mucositis. This suggested that the participants were motivated and dedicated to the completion of this investigation. The oral examinations were done independent of the patient-reported measures.

### Reporting of symptoms

**Figure 2:**
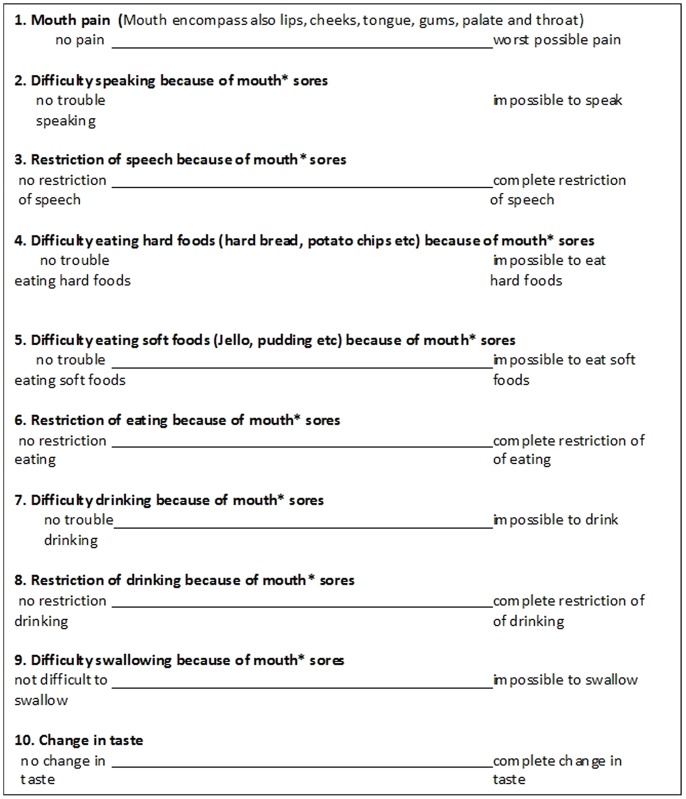
PROMS scale questionnaire with the ten components each detailing two extremes of a functional characteristic within a 100(VAS) (23).

The oral mucositis experience of the participants was assessed by using the PROMS scale [23]. The PROMS scale consists of 10, 100-mm horizontal visual analogue scales addressing oral functions affected by oral mucositis. Participants were asked to mark on the 100 mm line what best represented their present intra-oral condition (Fig. 2). During the baseline examination and prior to their completion of the actual PROMS scale questionnaire, participants were subjected to a few test-visual analogue scale questions focused on simple everyday topics to familiarize them with the concept of visual analogue scale assisted measurements. The participants completed a PROMS questionnaire at each clinical study appointment; baseline, twice per week during their radiotherapy period and at the post-operative visit, prior to and independently of the actual clinical oral examination.

### Data management and statistical analyses

A power analysis was done *a priori* to establish a rank correlation of rho = 0.90 between the PROMS scale and the NCI-CTCAE v.3 and/or OMAS scores and yielded a sample estimated size of 20 participants (Alpha level 0.05% and power of 80%, 2-tailed correlations) (Sample power, SPSS Inc. Chicago, USA). Since patients with H&N cancer may experience relatively high study dropout rates [25], it was considered prudent to recruit 50 participants into the study.

All recordings were documented using de-identified case report forms. The information from the case report forms was transferred into a relational database (MS Access, Microsoft Inc. Redmont, WA, USA). Repeated data entry verifications were made before exporting the data matrices for statistical analysis. The measured clinical and participant-reported variables were checked for normal distribution to establish a potential need for log-transformation corrections to obtain more precise p-values before being subjected to Spearman rank correlation. Spearman rank correlations were applied to characterize the relationships between the PROMS scale and the NCI-CTCAE v.3 as well as OMAS & TOTAL-VAS-OMAS scores using the statistical procedure “PROC CORR” in the SAS System Version 9.2 software (SAS Institute, Cary, NC, USA). To appraise the strengths of correlation at the different time points throughout the observation period robust repeated measures mixed linear models, “PROC MIXED”, were applied which account for the repeated nature of the measurements. Finally, a Bonferroni correction was applied to all statistical tests to account for multiple testing of the same measures. Correlations showing a Spearman's Rho of <0.20 were considered poor, 0.21–0.40 fair, 0.41–0.60 moderate, 0.61–0.80 good, and >0.80 very good [26].

## Results

Fifty patients were recruited and followed throughout radiation treatment between August 17, 2009 and July 19, 2010. During this time 520 clinical examinations were undertaken, of which 500 were undertaken by the first author (A.M.G.). Thirty-three participants completed the study, while 7 discontinued due to exhaustion. Ten participants either did not start or had stopped their cancer treatment (n = 7). Others were excluded because the prescribed radiation dose was below 54 Gray (n = 3). Most participants received radiation once daily for six (n = 7) or seven weeks (n = 25), while one participant received radiation twice daily for 4 weeks. Demographic information on participants who completed the study can be seen in (Table 1).

**Table 1:** Baseline Demographic and Clinical Characteristics of the participants who completed the full study (n = 33).

Characteristic	Subcategory	No. (%)
Sex	Male	25 (76)
	Female	8 (24)
Race	Caucasian	27 (82)
	Black	1 (3)
	Asian	5 (15)
Age (years) Mean (Standard deviation, Range)		61 (10, 38–78)
Dental status	Good	15 (45)
	Fair/Poor	16 (49)
	Edentulous	2 (6)
Smoking *	Never	9 (29)
	Present smoker	7 (22)
	Ex-smoker	16 (50)
Alcohol*	No	12 (38)
	Yes	20 (62)
Primary tumour location	Oral cavity/oropharynx	18 (55)
	Salivary glands	6 (18)
	Other	9 (27)
T stage	T0/TX	6 (18)
	T1	5 (15)
	T2	9 (27)
	T3	7 (21)
	T4	6 (18)
N stage	N0	15 (45)
	N1	5 (15)
	N2	12 (36)
	N3	1 (3)
Chemotherapy	No	18 (55)
	Yes	15 (45)
Therapy length	4 weeks	1 (3)
	6 weeks	7 (21)
	7 weeks	25 (76)

*1 unknown.

### Clinical signs and symptoms of oral mucositis

**Figure 3:**
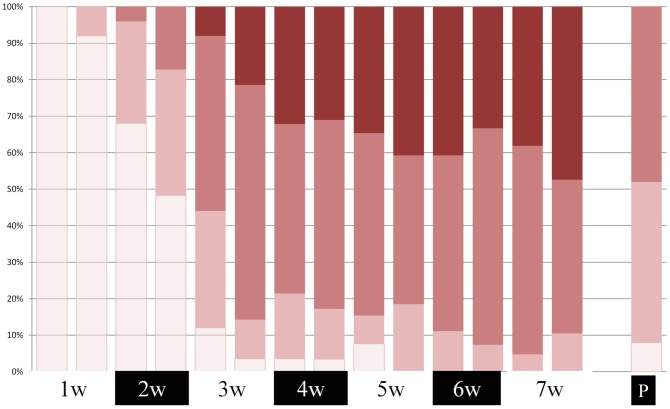
NCI-CTCAE v.3 (Cumulative %) (No color = Score 0, Dark = Score 3) recorded over the cancer treatment period (7 weeks) and at the 4–6 week post-therapy examination.

**Figure 4:**
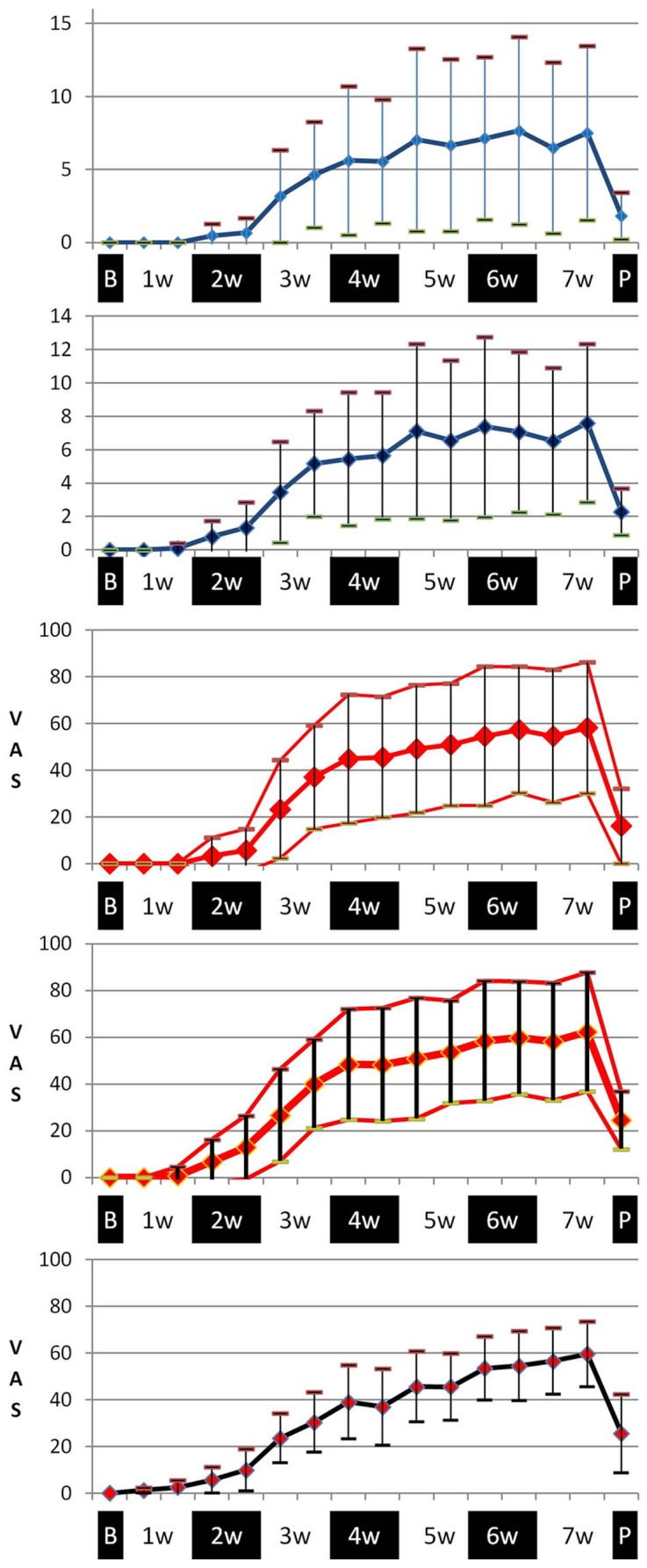
Clinical signs and patient symptoms recorded over the observation period (7 weeks) and at the 4–6 week post-therapy examination (“P”). From top to bottom: OMAS Scores for Ulceration (Means +/− SDs; maximum score = 27), OMAS Scores for Erythema (Means +/− SDs; maximum score = 18), TOTAL-VAS-OMAS Score for Ulceration (Means +/− SDs), TOTAL-VAS-OMAS Score for Erythema (Means +/− SDs) and PROMS scale value (Means +/− SDs). (All VAS scales: maximum value = 100).

NCI-CTCAE scores for oral mucositis of “1” were observed as early as the first week of cancer treatment, while scores of “3” started occurring towards the end of the second week. The prevalence of the score “3” was close to 50% by the end of the cancer treatment period (Fig. 3). This may be an underestimate as intra-oral scoring was not possible in some participants due to their inability or unwillingness to open their mouth for a complete clinical assessment. At the post treatment examination about 50% of the participants still demonstrated a NCI-CTCAE v.3 score of “2”. The OMAS-Ulceration and -Erythema as well as the TOTAL- VAS-Ulceration and -Erythema scores varied markedly amongst participants at the different time-points. However, the maximum scores were recorded consistently at the end of the 6–7 week fractionated radiotherapy period. At the post-treatment examination the average scores were approximately a third of the maximal scores reported during radiotherapy. The PROMS-aggregated scores increased gradually during cancer treatment period culminating with a visual analogue scale value of 60 by the end of treatment. Hence, all measurements displayed similar patterns of increasing oral mucositis scores with peaks at the end of cancer treatment. Signs and symptoms of oral mucositis were still present at the post-treatment examination carried out 4 to 6 weeks after ending cancer treatment (Fig. 4).

### Statistical correlations

**Figure 5:**
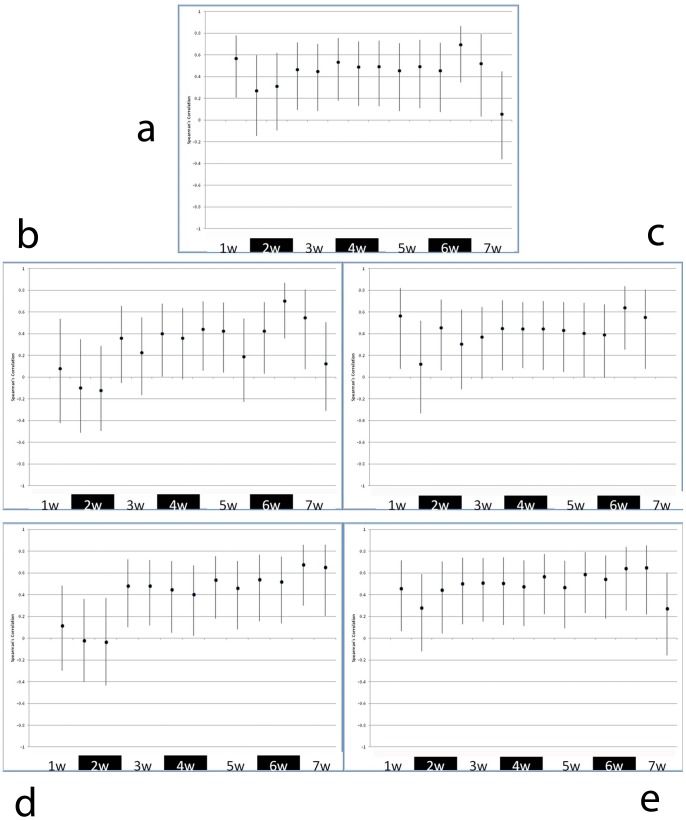
Spearman rho correlation coefficients over the observation period (7 weeks) and at the 4–6 week post-therapy examination between clinical signs of oral mucositis, as reported by different clinician-based scoring tools and the experience of oral mucositis by the participants, as reported by the PROMS scale. PROMS scale value vs. scores for: NCI-CTCAE v.3 (a), OMAS-Ulceration (b), OMAS Erythema (c), TOTAL-VAS-OMAS Ulceration (d) and TOTAL-VAS-OMAS Erythema (e).

The dataset used for statistical analyses was based on the 33 participants who completed the full study. The scorings of the 7 participants who discontinued the study did not appear to differ from the remaining up to the point of their drop-out. The normality of the data distribution of the measurement variables was checked for skewness before applying the Spearman rank correlation tests. Minimal skewness was observed, which enabled correlation analyses without log-transformation. Very good correlations (Spearman's rho 0.86–0.96) were observed between the different clinician-based scoring tools. Participant experience of oral mucositis using the PROMS scale demonstrated good correlations on the group level with clinician-determined scores over all the time points (Spearman's Rho 0.65–0.78, p<0.001). (Table 2). These correlations were performed over all time points using a statistical model that accounted for the repeated nature of the data assessment for calculation of p-values. PROMS scores for participant experience of oral mucositis demonstrated poor to good correlations on the group level at different time points with the clinician-determined scores. (Spearman's Rho -0.12–0.70, p<0.001). The correlations between PROMS scales and the scores obtained by measurement of clinical indices changed over time, but specific trends could not be established. At the critical phase where mouth opening was problematic, i.e., during the 6^th^ and 7^th^ week after commencing cancer treatment, the Spearman's Rho varied between 0.19 and 0.70 (p< 0.001) (Fig. 5).

**Table 2:** Spearman rho correlation coefficients over the full cancer treatment between clinical signs of oral mucositis, as reported by different clinician-based scoring tools and the experience of oral mucositis by the participants, as reported by the PROMS scale values.

	OMAS-U	OMAS-E	TOTAL-VAS-OMAS-U	TOTAL-VAS-OMAS- E	PROMS
NCI- CTCAE v. 3	0.89	0.86	0.92	0.91	0.75
OMAS Ulcerate Area	-	0.92	0.95	0.90	0.65
OMAS Erythema Area	-	-	0.91	0.92	0.69
TOTAL-VAS-OMAS Ulceration	-	-	-	0.96	0.75
TOTAL-VAS-OMAS Erythema	-	-	-	-	0.78

## Discussion

Oncology patients that undergo cancer treatment needs supporting care in time of extreme psychological duress [16]–[18]. Preventing and managing oral mucositis as a side-effect of the therapy is an important contribution to increase the patient endurance so he or she can tolerate and ultimately benefit from the cancer therapy. The combination of clinician-observed signs of oral mucositis and patient-reported experience of the symptoms of oral mucositis appears to be the best approach to assess the severity of oral mucositis, rather than relying exclusively on either one or the other. The current study shows that the PROMS scale can complement common clinician-determined assessments of oral mucositis. Moreover, the PROMS can also substitute the common clinician-determined assessments of oral mucositis in patients where these can't open their mouth or endure a comprehensive clinical oral examination or simply can't come to the treatment centre. There are several occasions when comprehensive clinical assessments of oral mucositis may be impossible, while data based on PROMS assessment can almost always be obtained. In these situations the PROMS score might be used to replace missing clinical data on an individual patient level. If needed, the PROMS questions can potentially be completed via telecommunications equipment (e.g. Internet) to substitute a clinical oral mucositis assessment during the cancer treatment.

It should be emphasized that the PROMS is not a measure of quality of life and does not address psychological duress, but is developed with an objective to elucidate the possible effectiveness of any therapeutic interventions against oral mucositis. To facilitate user-friendliness, only a limited number of questions are asked, and these focus on simple everyday daily functions that empirically are noted as side effects of radiotherapy. Including more questions is not necessarily advantageous, since completing the questionnaire will become more cumbersome for the patient. Admittedly, some questions may be redundant, which will be the focus of future studies. Moreover, including questions that would rely on adequate cognitive function such as enquiry about periodicity of burning sensations and incidence of bleeding would be unreliable due to the patients' extraordinary emotional circumstances [6].

A general impression was that few participants had any problems understanding the questions on the PROMS questionnaire relatively quickly. Moreover, completing the questionnaire was perceived by most as quick and easy and not felt as burdensome while they received their cancer treatments. If a patient-reporting instrument, such as the PROMS scale is implemented in routine care or as an outcome in a clinical trial it should be stressed to the patients in an early stage of their cancer treatment that the data generated from the PROMS may possibly become the only manner their intraoral oral mucositis status can be assessed at later stages in the cancer treatment period.

The correlation between the data using the PROMS and the clinician-based scoring tools were fairly similar over all the time periods as well as during the critical 6^th^ and 7^th^ weeks period during cancer treatment when the oral mucositis is at its worst. The extent of ulceration was not a sufficient indicator of the patient burdens experienced during the onset and development of oral mucositis. Yet, should not the patient burden direct changes in cancer treatment (ranging from reduction in the intensity of treatment to total cessation of treatment) as opposed to what might be less meaningful clinical assessments of lesion appearance and size? As with management of other chronic conditions characterized by pain, including ‘chronic pain’ itself, it is the patient burden that should ideally be used as the endpoint or outcome measure for making decisions regarding further treatment of the condition, in this case, oral mucositis. Thus, assessment of the extent of ulceration as the most important and in some cases sole outcome measure, while interesting and important from a mechanistic and pathophysiological point of view is important, it would seem much more critical to understand how a patient is functioning during cancer treatment, and the patient's relative extent of duress while undergoing the treatment so that appropriate measures can be taken. After all, a patient with ‘small’ areas of ulceration, but who is demonstrating severe levels of suffering, may require intervention, whereas a patient with larger ulcerations but minimal symptoms may not. This is something that simply cannot be measured by determining the size and/or extent of ulceration, particularly since the pain associated with this condition is considered complex and likely neuropathological in origin [19].

There was a large degree of heterogeneity in the participants of the study, particularly in relation to the location of their malignancy, tumour stage, sex and choice of radiotherapy procedure. (Table 1). Since the study was designed to test for the hypothesis that the patients' self-reported experience of oral mucositis correlated with other currently available measures of oral mucositis, the impact of this heterogeneity was considered to be of minor importance. There were no attempts to relate the oral mucositis data to any specific demographic, clinical or other extrinsic and intrinsic factors due to a high risk of spurious associations.

The three clinician-based scoring tools have different characteristics that need to be recognized. The clinical scoring of oral mucositis with the use of the ordinal NCI-CTCAE v.3 tool is in general straightforward, but borderline cases may be challenging to differentiate using this tool. In particular, the distinction between grades ‘2’ and ‘3’ can occasionally be challenging; a characteristic that reinforces subjectivity in making assessments. The challenge has apparently been recognized, since the NCI tools have undergone several modifications over the years in order to facilitate their use [24], [27]–[29].

Using the clinical component of the OMAS scale is also generally straightforward albeit more time consuming than using the NCI-CTCAE v. 3 tool [24]. A calibration booklet such as the one used in the current study facilitates scoring by visual comparison with photographs. A characteristic of the OMAS tool is that if severe oral mucositis is present in only one or two areas in the oral cavity but minimal or absent elsewhere, the total score for the severity of oral mucositis will be low, no matter how severely ulcerated those one or two areas are. The authors of the original paper outlined various ways of handling the sum-scores statistically, but ended up with more than one recommendation [21]. In light of the experiences of patients suffering from oral mucositis, it is uncertain whether having one area with severe erythema and/or ulcerations is worse than having multiple areas that on their own might be less severely involved. Moreover, it is not entirely clear why one large ulcer should affect the patient more (or not) than several small ulcerations.

The TOTAL-VAS-OMAS tool has so far only been tested by the developers in one patient population [23], and there are no published guideline documents regarding its use, challenges and interpretations. In the lack of pictorial guides or descriptors there is a possibility that observers, including the one in the current study may create skewed data since relatively high scores can be given in the early phases during cancer treatment before the really severe cases of oral mucositis become observable. Regardless of which clinician-based scoring tools is used, it is important that all sites of the oral cavity are examined, which can be difficult or uncomfortable at later stages of cancer treatment.

It is often tempting to interpret patient symptom data on inter-individual rather than on intra-individual levels. Self-assessed patients may enter a higher score than other patients depending on several factors including, but not limited to, previous experiences regarding illness or pain. Moreover, the number and strength of narcotic and non-narcotic analgesics could also affect self-reported experiences of mouth pain resulting from oral mucositis. Conversely, some participants continued to report significant mouth pain in spite of the use of high amounts of analgesic medication [30].

Many consider correlations between patients'-recorded subjective measures and clinically recorded measures obtained by health professionals as biased with high levels of variability. Attempts to minimize bias in the current study were done by undertaking calibration *a priori*, consistent use of a booklet/poster with clinical photographs while appraising the participants and use of mainly one single clinical examiner throughout the study. The great majority of the clinical examinations were completed by one investigator (A.M.G.), since one important factor for negative experiences in cancer trial participation is involving many physicians at check-up appointments [31]. Moreover, this assured that measurements were done mainly by one calibrated investigator which would tend to lead to less variability.Although there appears to be a statistical correlation between clinical signs and patient-reported symptoms on group level, multiple individuals deviated from this pattern in this study. Consequently, there is a possibility that subtle intra-individual improvements (or deteriorations) can be masked if the effectiveness of new therapeutic and preventive interventions targeted towards oral mucositis is determined on group level point estimates rather than on intra-individual levels.

## Conclusions

The current findings indicate good correlations between assessment of the oral mucositis experience obtained from the PROMS scale and currently available instruments used commonly to assess oral mucositis in patients with head and neck cancer. Hence, patient-based experiences of oral mucositis, as reported by the PROMS scale, may be a useful tool to augment clinical assessment of oral mucositis or as a substitute assessment in situations where patients cannot endure oral examinations.
